# Seroprevalence of hepatitis B e antigen (HBe antigen) and B core antibodies (IgG anti-HBcore and IgM anti-HBcore) among hepatitis B surface antigen positive blood donors at a Tertiary Centre in Nigeria

**DOI:** 10.1186/1756-0500-5-167

**Published:** 2012-03-28

**Authors:** Akinsegun A Akinbami, Olajumoke O Oshinaike, Owolabi A Dosunmu, Titilope A Adeyemo, Adewumi Adediran, Sulaiman Akanmu, Kikelomo O Wright, Seun Ilori, Kinsley Aile

**Affiliations:** 1 Department of Haematology and Blood Transfusion, Lagos State University College of Medicine, Ikeja, Nigeria; 2 Department of Internal Medicine, Lagos State University College of Medicine, Ikeja, Nigeria; 3 Department of Haematology and Blood Transfusion, Faculty of Clinical Sciences, College of Medicine, Idiaraba, Lagos, Nigeria; 4 Department of Community Health and Primary Health Care, Lagos State University College of Medicine, Ikeja, Nigeria; 5 APIN Clinic, Lagos University Teaching Hospital. Idiaraba, Lagos, Nigeria

**Keywords:** Seroprevalence, HBeAg, HBsAg, IgG anti-HBc, IgM anti-HBc, Blood donors

## Abstract

**Background:**

Hepatitis B virus (HBV) is a common cause of liver disease throughout the world. HBV is transmitted through blood and other body fluids, including semen and saliva. Chronic replication of HBV virons is characterized by persistence circulation of HBsAg, HBeAg and HBV DNA; usually with anti-HBc and occasionally with anti-HBs. Aim: To determine the prevalence of HBeAg, IgG anti-HBcore and IgM anti-HBcore amongst HBsAg positive blood donors. These parameters are reflective of transmissibility and active hepatitis B infection. A cross sectional study was carried out at the blood donor clinics of Lagos State University Teaching Hospital Ikeja and Lagos University Teaching Hospital Idiaraba. A total of 267 donors were recruited to determine HBe antigen, IgG and IgM anti-HBcore antibodies amongst hepatitis BsAg positive donors. Five milliliters of blood was collected from those who tested positive to HBsAg screen during donation. The sera were subjected to enzyme linked immunosorbent assay (ELISA). Pearson chi-squared test was used for the analytical assessment.

**Findings:**

A total number of 267 HBsAg positive blood donors were studied. A seroprevalence of 8.2% (22 of 267) HBeAg was obtained, 4 of 267 (1.5%) were indeterminate while 241 (90.3%) tested negative. Only 27 out of 267 donors (10.1%) tested positive to IgM anti-HBcore, 234(87.6%) tested negative, while 6(2.2%) were indeterminate. A higher percentage of 60.7% (162 of 267) tested positive to IgG anti-HBcore, while 39.3% (105 of 267) tested negative.

**Conclusion:**

There is a low seroprevalence rate of HBeAg-positive chronic hepatitis and relatively high IgG anti-HBcore and IgM anti-HBcore rates in South West Nigeria.

## Background

Hepatitis B virus (HBV) is a common cause of liver disease throughout the world. Cirrhosis, liver failure and hepatocellular carcinoma develop in 15–40% of chronically infected hepatitis B virus individuals [1]. HBV is transmitted through blood and other body fluids, including semen and saliva. The virus is hundred times more infectious than human immunodeficiency virus (HIV) and unlike HIV; it can live outside the body in dried blood for longer than a week [2].

It may present as acute hepatitis with resolution or chronic hepatitis which may evolve to cirrhosis and fulminant hepatitis with massive liver necrosis and the backdrop for hepatitis D virus infection. Chronic HBV infection is defined as hepatitis B surface antigen (HBsAg) positivity for at least six months [3]. Over 350 million of the 2 billion individuals infected with hepatitis B virus worldwide are chronically infected [4]. An estimated one third of the world’s population has serologic evidence of past infection and the virus causes more than one million deaths annually [5].

HBV infection occurs frequently in Nigeria [6,7]. It is estimated that about 12% of the total Nigerian population of 140 million is chronically infected with Hepatitis B virus [8,9].

The global prevalence of chronic hepatitis B infection varies widely, from >8% in Africa, Asia, and the Western Pacific to 2–7% in Southern and Eastern Europe, and to <2% in Western Europe, North America, and Australia. In the United States of America, an estimated 185,000 new infections evolved yearly [10].

Studies from different parts of Nigeria have reported varying prevalence rates among blood donors. A prevalence rate of 5.1% was found in blood donors in Ibadan, West [11], 11.4% in Zaria, North [12], 10.4% in Benin, South [13], 3.7% in Enugu, East [14], and 1.57% in Port Harcourt South [15]. Patients with chronic hepatitis represent carriers of actively replicating virus and hence are sources of infection to other individuals [16]. HBV also plays an important role in the development of hepatocellular carcinoma. The presence of only HBsAg neither indicates replication nor infectivity [17]. On the contrary, chronic replication of HBV virons is characterized by persistence circulation of HBsAg, HBeAg and HBV DNA; usually with anti-HBc and occasionally with anti-HBs [17], which may result to progressive liver damage in these patients [17]. The objective of this study was to determine the prevalence of HBeAg, IgG anti-HBcore and IgM anti-HBcore amongst HBsAg positive blood donors.

Although there is abundant data on the seroprevalence of HBsAg in Nigeria, there is need for more information on the seroprevalence of HBeAg, an indicator of hepatitis B virus transmissibility and HBcore antibodies which when present, indicates likely progression to liver cirrhosis, fulminant hepatitis and primary liver cell carcinoma. It is therefore important to study not only the seroprevalence of HBsAg amongst blood donors, but also to determine HBeAg and HBcore antibodies which determines infectivity and likelihood of progression amongst chronic carriers.

## Methods

### Study design

A cross sectional study was carried out at the blood donor clinics of Lagos State University Teaching Hospital Ikeja (LASUTH) and Lagos University Teaching Hospital Idiaraba (LUTH) between January 2010 and June 2011. Consent was obtained from each prospective blood donor recruited into the study and approval was obtained from both institutions’ Research and Ethics Committees. Participants completed structured questionnaires which included demographic information, previous history of blood transfusion, number of sexual partners, type of donor, history of unprotected sex, and use of intravenous drug. Assistance was rendered where necessary.

### Inclusion criteria

HBsAg positivity and all who met the criteria as blood donors i.e. age between 18–65 years, blood pressure <140/90 mmHg, pulse < 100 BPM, no blood donation in the last 3 months, no chronic illnesses such as diabetes mellitus, asthma, or history of allergy.

### Exclusion criteria

Those who did not meet the criteria for blood donation stated in the inclusion criteria and HBsAg negative donors.

### Collection of samples

Blood samples of 5 ml were collected from HBsAg-positive donors into sterile un-anticoagulated bottles and centrifuged. The sera were separated into other sterile bottles and stored at -20°C. Sera were tested for HBe antigen and HBcore IgG and IgM antibodies by the enzyme linked immunosorbent assay (ELISA). The hepatitis B-specific HBe antigen, HBcore IgG and IgM were studied by commercial DIA.PRO diagnostic Bioprobes ELISA kit (Italy) following manufacturer’s instruction. All specimens were analyzed using the enzyme immunoassay test. HBe antigen and HBcore IgM were interpreted as a ratio (S/Co) where S = optical density (OD) of the sample at 450 nm and Co is the cut off as follows < 0.9 = negative, 0.9–1.1 = equivocal, >1.1 = Positive. While HBcore IgG was interpreted as a ratio of the cut off and sample optical density (OD) of 450 nm (Co/S) as follows; < 0.9 = negative, 0.9–1.1 = equivocal, >1.1 = Positive. The controls and calibrators passed the validation check recommended by the manufacturers of the three kits.

### Sample size determination

Most studies in Nigeria reported average seroprevalence rates of 10% [18-20]. This figure was used to calculate the sample size for this study using the formula [21] n = z^2^pq/d^2^ where Z = 1.96 normal deviate representing the 95% confidence limit, d = 0.05 as acceptable margin of error, p = reported seroprevalence 10% (0.1), q which is the probability of the event not occurring, which will be 1-p = 0.9 to obtain a minimum sample size of 138.22. However, a sample size of 267 was used to determine the true seroprevalence.

### Statistical assessment

A statistical software package SPSS version 16 was used for data analysis. The descriptive data were given as means ± SD. The chi-squared was used for the analytical assessment. The differences were considered to be statistically significant when the p value obtained was < 0.05.

## Findings

A total of 267 HBsAg positive blood donors were studied. The mean age was 33.73 ±7.988 years with a minimum age of 17 years and maximum of 59 years. Majority, 261 of 267 donors (97.8%) were males, while 6 of 267 (2.2%) were females (Table 1). The donors consisted predominantly of family replacement donors which number was 237of 267 of donors (88.8%), while 30 of the 267 donors (11.2%) were voluntary donors. Only 114 (42.7%) were single and 153 of 267 (57.3%) were married. A total of 265 of 267 (99.3%) of the donors gave history of having only one sexual partner, while 1 (0.4%) each had two and three sexual partners. All the participants were heterosexual and gave no history of previous sexually transmitted infection. A total of 64 (24%) of the donors had never had unprotected sex, 190 (71.2%) occasionally had unprotected sex, and 13(4.9%) frequently engaged in unprotected sex. Only 11(4.1%) admitted to use of intravenous drug, 83 of the 267 (31.1%) had previous history of blood transfusion while 184 (68.9%) gave no previous history of blood transfusion. Majority of the donors 253(94.8%) gave no previous history of jaundice, while 14 (5.2%) had a previous history of jaundice.

**Table 1:** Donors’ parameters

	**Frequency**	**Percent**
**Sex**		
Male	261	97.8%
Female	6	2.2%
**Total**	267	100%
**Marital status**		
Married	153	57.3%
Single	114	42.7%
**Total**	267	100%
**Past Blood Transfusion**		
Yes	83	31.1%
No	184	68.9%
**Total**	267	100%
**Types of Donor**		
Voluntary	30	11.2%
Family replacement	237	88.8%
**Total**	267	100%

A seroprevalence of 8.2% (22 of 267) HBe Ag- positive donors was obtained (Table 2), 4 of the 267 (1.5%) donors were indeterminate while 241(90.3%) tested negative. Only 27 of the 267 (10.1%) tested positive to IgM anti-HBcore, 234(87.6%) tested negative, while 6(2.2%) were indeterminate. A higher percentage of 60.7% (162 of 267) tested positive to IgG anti-HBcore, and 39.3% (105 of 267) tested negative.

**Table 2:** Seroprevalence of HBeAg, IgG anti-HBcore and IgM anti-HBcore

	**Frequency**	**Percent**
**HBe Ag**		
Negative	241	90.3%
Positive	22	8.2%
Indeterminate	4	1.5%
**Total**	267	100%
**IgG anti-HBc**		
Negative	105	39.3%
Positive	162	60.7%
**Total**	267	100%
**IgM anti-HBc**		
Negative	234	87.6%
Positive	27	10.1%
Indeterminate	6	2.2%
**Total**	267	100%

Out of the 22 donors who tested positive to HBeAg, and 4 that were indeterminate, 10 (38.5%) had previous history of blood transfusion, while 16 (61.5%) had no history of blood transfusion p value = 0.666. (Table 3). Only 2(7.7%) were voluntary blood donors, while 24(92.3%) were replacement donors p value = 0.208. Majority 18(69.2%) admitted to occasional unprotected sex, only 1 (3.8%) always had unprotected sex, while 7(26.9%) never had unprotected sex. P value = 0.504. A total of 20 (76.9%) of 26 of these subjects, tested positive to IgG anti-HBcore, while 6 (23.1%) tested negative. Only 4(15.4%) tested positive to IgM anti-HBcore, majority 21 (80.8%) tested negative, while 1 (3.8%) was indeterminate.

**Table 3:** Parameters of subjects HBe Ag positive

	**Frequency**	**Percent**
**Previous Blood Transfusion**		
Yes	10	38.5%
No	16	61.5%
**Total**	26	100% P=0.666
**Type of donors**		
Voluntary	2	7.7%
Replacement	24	92.3%
**Total**	26	100% P=0.208
**IgGanti-HBcore**		
Negative	6	23.1%
Positive	20	76.9%
**Total**	26	100%
**IgM anti-HBcore**		
Negative	21	80.8%
Positive	4	15.4%
Indeterminate	1	3.8%
**Total**	26	100%

Almost all 240 of the 241 (99.6%) who tested negative to HBeAg had one sexual partner, 73 of the 241(30.3%) had been transfused with blood, while 168 of the 241 (69.7%) had no past history of blood transfusion. Out of the total number of 267 enrolled for the study, almost all (28 of 30) voluntary donors (10.4%) tested negative to HBeAg while 88.6% replacement donors tested negative. Similarly, out of the 241 who tested negative to HBeAg, 142 of 241(58.9%) tested positive to HBcore IgG and 99 of the 241(41.1%) tested negative.

## Discussion

Low seroprevalence rates of HBeAg-positive hepatitis were reported in various parts of Nigeria. The value of 8.2% obtained in this study is similar to 8.6% reported at Enugu city, East [18], 10.8% at Ibadan, West [19], and 8.8% at Benin City, South [20].

Chronic hepatitis is characterized by either the presence or absence of HBeAg. A significant proportion of this study population (91.8%) was HBeAg- negative. Similarly high values of 86.4% was obtained in Italy, [22] the Mediterranean, (30–80%) [23] Asia and in the Far East [24]. This form of chronic HBV HBeAg-negative CHB is common in areas where infection occurs in early childhood [25]. HBeAg-negative patients harbor HBV variants with a mutation in the core promoter or the pre core region of the HBV genome therefore affecting production of HBeAg but not replication [23]. The most frequent variant creates a stop codon in the precore region which completely abolishes the production of HBeAg [26]. The proportion of HBeAg-negative is rising worldwide, with patients with HBeAg-negative chronic hepatitis developing more active, advanced, and progressive liver disease. The consequent severe necro-inflammation is associated with infection in over 50% of the patients [27]. Chronic HBeAg-negative hepatitis is usually a late phase in the natural history of chronic HBV infection, rather than a result of de novo infection with a mutated variant [27].

A seroprevalence of 60.7% of IgG anti-HB core was obtained amongst the HBsAg positive blood donors in this study. Practically all HBsAg positive are expected to be IgG anti-HBc positive. This could be a reflection of the high false positive rate of the kit used for HBsAg screening at study centres. A 4^th^ generation ELISA kit manufactured by Bio-Rads laboratories (France) are used routinely for HBsAg positive screening amongst blood donors at both centres. The kit has a high sensitivity and possibly a high false positive rate with the aim of protecting recipient from infection. While 10.1% had IgM anti-HBcore which signifies active and recent infection. Sheik et al. had reported a seroprevalence of 10% of IgM anti-HBcore in Pakistan [28]. A 4% seroprevalence of IgM anti-HBcore was reported in Italy (a low prevalence area) amongst HBsAg positive donors who were chronically infected but 85% of the same patients tested positive to IgM anti-HBcore when they were acutely infected [29].

A total (consisting of both IgG anti-HBcore and IgM anti-HBcore) anti HBcore of 16.2% was reported in Yemen [30], and 13.5% in Korea [31], amongst blood donors. Antibody to hepatitis B core antigen is the first to develop, following acute hepatitis B infection, which appears predominantly as IgM anti-HBc at about 6 weeks after infection. The antibody typically persists for life except resolution of acute hepatitis B virus infection occurs. However, a significant proportion of hepatitis B surface antigen negative of the population in high prevalence areas like Nigeria, may test positive to anti-HBc, these individuals are described as having isolated anti-HBc hepatitis. It is not clear whether patients with isolated hepatitis are infectious; however, HBV DNA has been detected in the serum of patients with isolated anti-HBc hepatitis. Antar et al. reported a 7.8% prevalence of isolated hepatitis amongst Egyptian blood donors, 6.25% of these patients had detectable serum HBV DNA [32]. The issue of isolated hepatitis brings to forefront the ineffectiveness of HBsAg screening (which is what is used in Nigeria) in protecting blood recipients from hepatitis B infection. It has been suggested that anti-HBc testing should be combined with HBsAg screening of blood donors to reduce the risk of HBV infection [33].

This study could not establish a significant association between chronic hepatitis and previous history of blood transfusion, and also whether the donor was a voluntary or replacement donor, or the issue of having unprotected sexual intercourse the three major risk factors of infectivity. Majority of the individuals in areas with high prevalence of chronic hepatitis B infection like Nigeria where carrier rates are >5% are infected at birth or during early childhood [34]. Hence majority of the patients could have acquired the infection at birth or early childhood.

Hepatitis B vaccination for newborn babies was introduced to Nigeria about ten years ago as part of the expanded programme on immunization, the vaccination offers >95% protection against the development of chronic hepatitis infection [34]. None of the blood donors used in this study could have benefitted from the hepatitis B vaccination, because the minimum age of the blood donors in the study was 17 years.

## Conclusion

A low seroprevalence rate of 8.2% HBeAg-positive and high 91.8% HBeAg-negative chronic hepatitis and a relatively high IgG anti-HBcore and IgM anti-HBcore is seen the Nigeria.

## Competing interests

The authors declare that they have no competing interest.

## Authors’ contributions

AA-Conceptualized and designed the study. OO- Drafted the manuscript. OA- Reviewed the manuscript. AT- Acquisition of data. AA- Data analysis. AS-General supervision KO- Reviewed the final manuscript. SI-Carried out the ELISA. KI –Collected the samples. All authors read and approved the final manuscript.
